# Catalogue of the Howard University

**Published:** 1869-08

**Authors:** 


### Catalogue of the Howard University.

We have received a catalogue of this institution now organized in all the departments of a university. In 1867 Congress passed an act incorporating the Howard University to consist of Normal, Collegiate, Theological, Law, Medical, Agricultural, and such other departments as its board of Trustees should establish.

The Trustees have elected to the various professorships in the Medical Department very worthy and efficient men. We are pleased to see that Dr. Phineas H. Strong of our city has received the honor of appointment to one of the most important chairs in the Medical Department—Professor of the Principles and Practice of Medicine. We most heartily congratulate the University upon being able to se cure the services of so capable and worthy a teacher.

